# Immunoscore encompassing CD3+ and CD8+ T cell densities in distant metastasis is a robust prognostic marker for advanced colorectal cancer

**DOI:** 10.18632/oncotarget.13207

**Published:** 2016-11-08

**Authors:** Yoonjin Kwak, Jiwon Koh, Duck-Woo Kim, Sung-Bum Kang, Woo Ho Kim, Hye Seung Lee

**Affiliations:** ^1^ Department of Pathology, Seoul National University Bundang Hospital, Seongnam, South Korea; ^2^ Department of Pathology, Seoul National University College of Medicine, Seoul, South Korea; ^3^ Department of Surgery, Seoul National University Bundang Hospital, Seongnam, South Korea

**Keywords:** colorectal cancer, tumor-infiltrating lymphocytes, tumor-associated macrophage, immunoscore

## Abstract

**Background:**

The immunoscore (IS), an index based on the density of CD3^+^ and CD8^+^ tumor-infiltrating lymphocytes (TILs) in the tumor center (CT) and invasive margin (IM), has gained considerable attention as a prognostic marker. Tumor-associated macrophages (TAMs) have also been reported to have prognostic value. However, its clinical significance has not been fully clarified in patients with advanced CRC who present with distant metastases.

**Methods:**

The density of CD3^+^, CD4^+^, CD8^+^, FOXP3^+^, CD68^+^, and CD163^+^ immune cells within CRC tissue procured from three sites–the primary CT, IM, and distant metastasis (DM)–was determined using immunohistochemistry and digital image analyzer (n=196). The IS was obtained by quantifying the densities of CD3^+^ and CD8^+^ TILs in the CT and IM. IS-metastatic and IS-macrophage–additional IS models designed in this study–were obtained by adding the score of CD3 and CD8 in DM and the score of CD163 in primary tumors (CT and IM), respectively, to the IS.

**Result:**

Higher IS, IS-metastatic, and IS-macrophage values were significantly correlated with better prognosis (p=0.020, p≤0.001, and p=0.005, respectively). Multivariate analysis revealed that only IS-metastatic was an independent prognostic marker (p=0.012). No significant correlation was observed between *KRAS* mutation and three IS models. However, in the subgroup analysis, IS-metastatic showed a prognostic association regardless of the *KRAS* mutational status.

**Conclusion:**

IS is a reproducible method for predicting the survival of patients with advanced CRC. Additionally, an IS including the CD3^+^ and CD8^+^ TIL densities at DM could be a strong prognostic marker for advanced CRC.

## INTRODUCTION

Tumor-infiltrating immune cells can influence tumor progression and metastasis. While one of their functions is recognition and elimination of tumor cells [1], they have also been reported to promote immune evasion by tumor cells and, eventually, metastasis [2–4]. Recent reports suggest that tumor-infiltrating lymphocytes (TILs) have an important role in boosting anti-tumor immunity against CRC [5–9] and other malignancies [10–15]. However, like other components of the tumor microenvironment, TILs display heterogeneity in their target site [8, 16]. This heterogeneity causes difficulties in determining their roles.

Recently, several studies have demonstrated that TILs have high prognostic utility. Galon et al. introduced the ‘immunoscore (IS)’, a value based on the density of CD3^+^ and CD8^+^ lymphocytes in the tumor center (CT) and invasive margin (IM) [17–19]. Moreover, some authors have reported that the IS method is superior to the current tumor-node-metastases (TNM) staging system, especially in colon cancers [20, 21]. However, the evidence is limited to stages I–III of the disease [18, 22].

Tumor-associated macrophages (TAMs) are another component of tumor-infiltrating immune cells. Macrophages are derived from monocytes and exhibit two polarization states in response to different microenvironmental signals–M1 and M2 [23–25]. M1 macrophages are pro-inflammatory and function as bactericides and antigen-presenting cells. M2 macrophages have an immunosuppressive phenotype. Several studies have revealed that M2 macrophage infiltration is associated with unfavorable outcomes in patients with CRC [26–29]. However, other studies have revealed that high infiltration by M1 as well as M2 macrophages is correlated with good prognosis [30]. Therefore, the prognostic utility of TAMs remains unclear.

The aim of this study was to confirm the prognostic value of the IS in patients with advanced CRC. The characteristics of tumor infiltrating immune cells was also determined. Additionally the heterogeneity in the target sites of tumor-infiltrating immune cells in patients with advanced CRC was evaluated.

## RESULTS

### The heterogeneous density of tumor infiltrating immune cells according to tumor location

**Figure 1:**
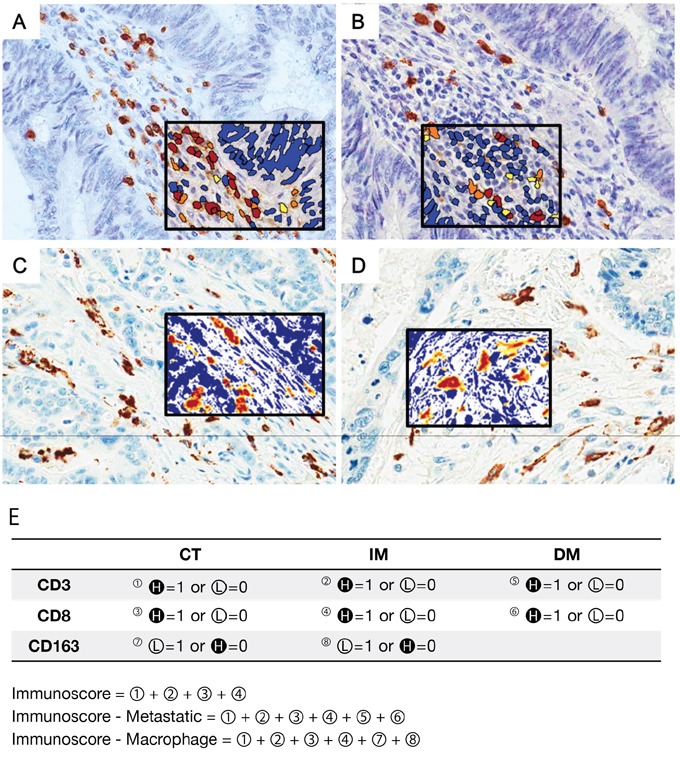
Representative figures of immunohistochemistry for tumor-infiltrating immune cells (×400) and schematic description of the immunoscore (IS) model Tumor-infiltrating lymphocytes (TILs) were stained with CD3 **(A)** and CD8 **(B)** antibodies. Tumor-associated macrophages (TAMs) were confirmed using CD68 **(C)** and CD163 **(D)** antibodies. The density of each subset of immune cells was counted by an image analysis system. The black squared inset presents the results of image analysis. The immunostained area is shown in red, and the non-immunostained area is shown in blue. **E.** The IS model is based on the enumeration of two lymphocyte subsets (CD3 and CD8) in the CT and IM of the primary tumor. All patients were grouped into high-density (H in dark circle) and low-density (L in light circle) groups for each marker in each region. The IS-metastatic (IS-M) model additionally includes lymphocyte density data in distant metastases. In the IS-macrophage (IS-ma) model, data of CD163^+^ macrophage density in the CT and IM were added. Because our data showed that TAMs had an opposite prognostic correlation compared to that of TILs, a low density of TAMs and a high density of TILs was recorded as a score.

**Figure 2:**
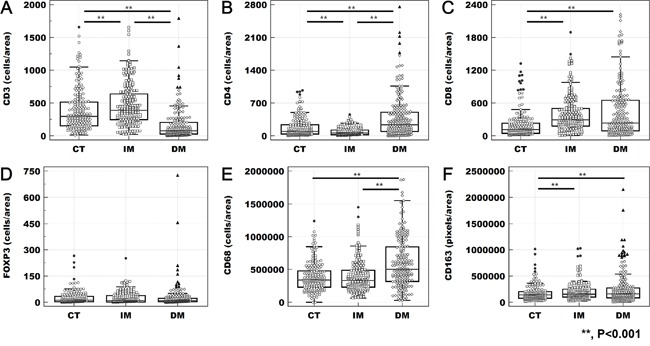
Heterogeneity of tumor-infiltrating immune cells The density of CD3^+^ **(A)**, CD4^+^ **(B)**, CD8^+^ **(C)**, CD68^+^ **(E)**, and CD163^+^ **(F)** cells differed significantly according to tumor location (**; p < 0.001 in the results of the paired *t*-test). However, the density of FOXP3^+^ lymphocytes **(D)** did not present heterogeneity according to tumor location.

Representative results of immunohistochemistry for tumor infiltrating immune cells are shown in Figure 1. The cell count per area (cells/mm2) of CD3^+^ lymphocyte was the highest in the IM (median, interquartile range (IQR); 389.15, 246.95–649.42) than any other site (297.79, 154.13–516.33 at the CT; 76.27, 28.04–204.55 at the DM). The density of CD8^+^ lymphocytes was lower in the CT (112.24, 48.42–232.98) than the IM (293.20, 177.85–504.41) and the DM (235.68, 91.52–648.20). The pixel count per area (pixels/mm2) of CD68^+^ macrophages was the highest in the DM (500631.05, 318786.38–844905.83). Similarly, CD163-positive macrophages were more frequently infiltrating in the DM (160636.11, 85120.41–283752.28) than any other site. All tumor-infiltrating immune cells except FOXP3^+^ lymphocytes presented a heterogeneous density according to tumor location (Figure 2). The comprehensive median and IQR values of the density of each tumor's microenvironmental factors are described in Table 1 .

**Table 1:** The median, IQR, and cut-off values of the tumor-infiltrating immune cells

		Median	IQR	Cut-off value
**CD3**	Tumor center	297.79	154.13 - 516.33	158.52
	Invasive margin	389.15	246.95 - 649.42	321.15
	Distant metastasis	76.27	28.04 - 204.55	272.23
**CD4**	Tumor center	98.01	38.30 - 241.05	22.88
	Invasive margin	59.01	24.03 - 124.82	82.25
	Distant metastasis	238.52	94.42 - 506.93	52.61
**CD8**	Tumor center	112.24	48.42 - 232.98	310.10
	Invasive margin	293.20	177.85 - 504.41	164.67
	Distant metastasis	235.68	91.52 - 648.20	98.92
**FOXP3**	Tumor center	11.67	2.92 - 33.33	6.37
	Invasive margin	9.58	2.52 - 38.26	0.71
	Distant metastasis	9.36	2.72 - 23.27	33.69
**CD68**	Tumor center	340080.87	229761.65 - 480635.88	623734.20
	Invasive margin	330204.65	233509.22 - 485385.06	278123.90
	Distant metastasis	500631.05	318786.38 - 844905.83	488839.60
**CD163**	Tumor center	138787.44	80061.73 - 201969.89	328155.00
	Invasive margin	153225.53	100686.21 - 250086.39	230371.20
	Distant metastasis	160636.11	85120.41 – 273752.28	53170.20

**Figure 3:**
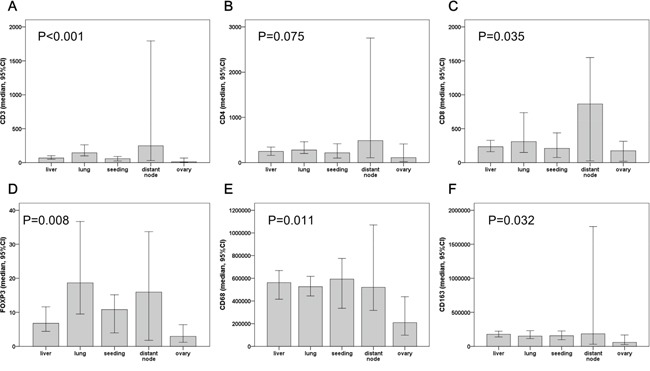
The density of tumor infiltrating immune cells at different distant metastasis sites CD3^+^ **(A)**, CD4^+^ **(B)** and CD8^+^ **(C)** lymphocytes were more frequently observed in non-regional lymph nodes. However, FOXP3^+^ lymphocytes **(D)** were higher in lung metastases than in distant lymph nodes. Including CD68^+^ **(E)** and CD163^+^ **(F)** macrophages, all tumor-infiltrating immune cells have the lowest density in ovary metastases.

The density of each tumor-infiltrating immune cell was varies in relation to the organ of metastasis (Figure 3). CD3^+^, CD4^+^, and CD8^+^ lymphocytes are denser in metastatic non-regional lymph node than in any other metastatic site. However, FOXP3^+^ lymphocytes are observed more frequently in lung metastases than in distant metastatic lymph nodes. All marker-positive immune cells had the lowest density in the ovary.

### Prognostic correlation of tumor-infiltrating immune cells in advanced CRCs

We divided the patients into low and high groups by the predetermined cut-off values of the continuous variables using maximal chi square method according to each immune cell marker in each tumor location. The Kaplan-Meier method revealed that a low density of CD3^+^ lymphocytes in the CT and the DM was statistically associated with a poor outcome (p = 0.030 and p = 0.013, respectively). A low density of CD4^+^ lymphocytes in the CT and IM was also related to a poor outcome (p = 0.001 and p = 0.018, respectively). In contrast, there was an association between CD8^+^ and FOXP3^+^ lymphocytes in the DM and patient worse outcome (p = 0.002 and p = 0.008, CD8 and FOXP3 respectively). The patients presenting with a high density of CD68^+^ and CD163^+^ macrophages in the CT of their primary tumor had significantly worse outcomes. Additional data on the median survival time and comparisons between patient groups according to immune cell markers and respective tumor location are listed in Supplementary Table 1.

### Application of immunoscore and their clinical implications in advanced CRCs

Through the assembled density data, we evaluated the patients with the IS system, which gives a score depending on the total number of high densities marked (from IS0 to IS4). Owing to loss of tissue microarray (TMA) core tissue, IS results were available for only 193 of 196 patients. According to the IS, 49.7% (96/193) was recorded as a low IS (IS0: 5.7%, IS1: 19.7% and IS2: 24.4%) and 50.3% (96/193) was a high IS (IS3: 43.0% and IS4: 7.3%).

In the present study, we used two additional scoring models that incorporate the results of macrophage infiltration into the primary tumor (IS-macrophage, IS-ma) and lymphocyte infiltrates into distant metastases (IS-metastatic, IS-M). IS-ma and IS-M results were available in 193 and 188 patients, respectively. Seventy five (38.9%) patients were low IS-ma (IS-ma0: 0%, IS-ma1: 1.0%, IS-ma2: 10.9%, and IS-ma3: 26.9%) and 118 (61.1%) patients presented with a high IS-ma (IS-ma4: 28.0%, IS-ma5: 29.0%, and IS-ma6: 4.1%). Of 188 patients, 53.2% (100/188) had low IS-M (IS-M0: 3.2%, IS-M1: 10.6%, IS-M2: 16.0%, and IS-M3: 23.4%) and 46.8% (88/100) had high IS-M (IS-M4: 33.5%, IS-M5: 10.6%, and IS-M6: 2.7%).

When the IS was compared to the patient's clinicopathologic features, higher pT stage (p = 0.001) and the presence of perineural invasion (p = 0.008) were significantly correlated with lower IS (Table 2). Lower IS-ma was associated with higher pT stage (p = 0.004) and synchronous metastasis (p = 0.007). Lower IS-M was also correlated with aggressive clinicopathological features, including higher pT stage (p < 0.001), lymphatic invasion (p = 0.003), and perineural invasion (p = 0.004).

**Table 2:** Clinicopathologic factors and tumor-infiltrating immune cells

Characteristics	Immunoscore			Immunoscore-macrophage			Immunoscore-metastatic		
	Low (0-2) (%)	High (3-4) (%)	P	Low (0-3) (%)	High (4-6) (%)	P	Low (0-3) (%)	High (4-6) (%)	P
Age (mean±SD)	60.41±1.33	59.38±1.19	0.459	60.89±1.52	59.25±1.09	0.308	60.11±1.31	59.66±1.20	0.801
Gender			0.666			0.767			0.559
Male	50 (52.1)	54 (55.7)		39 (52.0)	65 (55.1)		52 (52.0)	50 (56.8)	
Female	46 (47.9)	43 (44.3)		36 (48.0)	53 (44.9)		48 (48.0)	38 (43.2)	
pT stage			0.001			0.004			<0.001
pT1-3	47 (49.0)	71 (73.2)		36 (48.0)	82 (69.5)		49 (49.0)	65 (73.9)	
pT4	49 (51.0)	26 (26.8)		39 (52.0)	36 (30.5)		51 (51.0)	23 (26.1)	
pN stage			0.738			0.570			0.083
pN0	17 (17.7)	19 (19.6)		12 (16.0)	24 (20.3)		14 (14.0)	21 (23.9)	
pN1-2	79 (82.3)	78 (80.4)		63 (84.0)	94 (79.7)		86 (86.0)	67 (76.1)	
Metastasis			0.143			0.007			0.087
Metachronous	28 (29.2)	38 (39.2)		15 (20.0)	46 (39.0)		29 (29.0)	36 (40.9)	
Synchronous	68 (70.8)	59 (60.8)		60 (80.0)	72 (61.0)		71 (71.0)	52 (59.1)	
Lymphatic invasion			0.104			0.119			0.003
Absent	27 (28.1)	38 (39.2)		20 (26.7)	45 (38.1)		24 (24.0)	39 (44.3)	
Present	69 (71.9)	59 (60.8)		55 (73.3)	73 (61.9)		76 (76.0)	49 (55.7)	
Perineural invasion			0.008			0.240			0.004
Absent	37 (38.5)	56 (57.7)		32 (42.7)	61 (51.7)		39 (39.0)	53 (60.2)	
Present	59 (61.5)	41 (42.3)		43 (57.3)	57 (48.3)		61 (61.0)	35 (39.8)	
KRAS mutation			1.000			0.103			1.000
Absent	45(46.9)	44 (45.8)		29 (38.7)	60 (51.3)		46 (46.0)	41 (46.6)	
Present	51 (53.1)	52 (54.2)		46 (61.3)	57 (48.7)		54 (54.0)	47 (53.4)	
PIK3CA mutation			0.679			0.517			1.000
Absent	82 (85.4)	85 (87.6)		63 (84.0)	104 (88.1)		87 (87.0)	76 (86.4)	
Present	14 (14.6)	12 (12.4)		12 (16.0)	14 (11.9)		13 (13.0)	12 (13.6)	
BRAF mutation			0.065						0.123
Absent	90 (93.8)	96 (99.0)		71 (94.7)	115 (97.5)	0.434	94 (94.0)	87 (98.9)	
Present	6 (6.3)	1 (1.0)		4 (5.3)	3 (2.5)		6 (6.0)	1 (1.1)	
Total	96(100.0)	97 (100.0)		75 (100.0)	118 (100.0)		100 (100.0)	88 (100.0)	

### Prognostic value of immunoscore models in advanced CRCs

**Figure 4:**
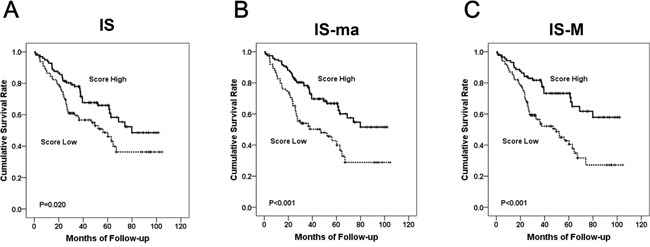
The Kaplan Meier survival curve according to each IS model The IS **(A)**, IS-ma **(B)**, and IS-M **(C).**

The Kaplan-Meier analysis revealed that all three IS models had a prognostic association. Higher scores were significantly correlated with improved survival (p = 0.021, p < 0.001, and p < 0.001, for IS, IS-ma, and IS-M, respectively) (Figure 4). By univariate COX regression analysis, the hazard ratios of IS, IS-ma, and IS-M were 1.666, 2.165, and 2.431, respectively (Table 3). Among other clinicopathologic features, age, advanced pT and pN stage, synchronous metastasis, lymphatic invasion, and perineural invasion were correlated with poorer outcomes.

**Table 3:** Univariate analysis according to clinicopathologic features including IS models

Variables	Univariate survival analysis	
	HR (95% CI)	P value
Age (≥65 vs. <65)	1.680 (1.096-2.575)	0.017
pT stage (T4 vs. T1-3)	2.256 (1.468-3.466)	<0.001
pN stage (N1-2 vs. N0)	4.186 (1.919-9.131)	<0.001
Metastasis (Synchronous vs. Metachronous)	4.407 (2.421-8.023)	<0.001
Differentiation (Poorly to Undifferentiated vs. Well to moderately differentiated)	1.744 (0.998-3.049)	0.051
Lymphatic invasion (Present vs. Absent)	2.889 (1.653-5.051)	<0.001
Perineural invasion (Present vs. Absent)	2.510 (1.591-3.962)	<0.001
Venous invasion (Present vs. Absent)	1.204 (0.757-1.913)	0.433
Immunoscore (Low (score 0-2) vs. High (score 3-4))	1.666 (1.079-2.572)	0.021
Immunoscore-macrophage (Low (score 0-3) vs. High (score 4-6))	2.165 (1.408-3.328)	<0.001
Immunoscore-metastatic (Low (score 0-3) vs. High (score 4-6))	2.431 (1.527-3870)	<0.001

Multivariate COX regression analysis revealed that of all three IS models, only the IS-M model was an independent prognostic factor (p = 0.012) (Table 4). Older age and synchronous metastases were also independent prognostic factors. The hazard ratio of a low IS-M was 1.858, higher than that of advanced pT and pN stage (1.291 and 1.874, respectively).

**Table 4:** Multivariate analysis according to clinicopathologic features including IS models

Score model	Variables	Multivariate survival analysis	
		HR (95% CI)	P value
A	**Immunoscore (Low (score 0-2) vs. High (score 3-4))**	1.336 (0.852-2.094)	0.206
	Age (≥65 vs. <65)	2.160 (1.374-3.394)	0.001
	pT stage (T4 vs. T1-3)	1.229 (0.779-1.940)	0.375
	pN stage (N1-2 vs. N0)	1.892 (0.849-4.219)	0.119
	Metastasis (Synchronous vs. Metachronous)	3.677 (1.927-7.016)	<0.001
	Lymphatic invasion (Present vs. Absent)	1.958 (1.095-3.502)	0.023
	Perineural invasion (Present vs. Absent)	1.448 (0.896-2.339)	0.131
B	**Immunoscore-macrophage (Low (score 0-3) vs. High (score 4-6))**	1.525 (0.981-2.370)	0.061
	Age (≥65 vs. <65)	1.031 (1.011-1.052)	0.002
	pT stage (T4 vs. T1-3)	1.252 (0.794-1.973)	0.333
	pN stage (N1-2 vs. N0)	2.071 (0.933-4.598)	0.074
	Metastasis (Synchronous vs. Metachronous)	3.402 (1.796-6.447)	<0.001
	Lymphatic invasion (Present vs. Absent)	2.021 (1.129-3.615)	0.018
	Perineural invasion (Present vs. Absent)	1.537 (0.956-2.470)	0.076
C	**Immunoscore-metastatic (Low (score 0-3) vs. High (score 4-6))**	1.858 (1.144-3.018)	0.012
	Age (≥65 vs. <65)	2.359 (1.477-3.766)	<0.001
	pT stage (T4 vs. T1-3)	1.291 (0.814-2.048)	0.278
	pN stage (N1-2 vs. N0)	1.874 (0.847-4.146)	0.121
	Metastasis (Synchronous vs. Metachronous)	3.696 (1.935-7.060)	<0.001
	Lymphatic invasion (Present vs. Absent)	1.744 (0.975-3.122)	0.061
	Perineural invasion (Present vs. Absent)	1.422 (0.881-2.296)	0.149

### Relationship of mutational status with immune cell infiltration and IS

Of the 196 cases examined, 89 (45.6%) had wild-type *KRAS* and 106 (54.4%) had mutated *KRAS*. Among the tumors with mutated *KRAS*, mutations in codon 12 or 13 were identified in 99 (93.4%). Additionally, mutations in *BRAF* (V600E) were identified in 7 patients (3.6%), and those in *PIK3CA* were identified in 25 patients (13.1%). The two most common *PIK3CA* mutations were found in exon 9 (17 cases, 68.0%) and exon 20 (5 cases, 20.0%).

There was no difference in the T cell densities of tumors with *KRAS* or *PIK3CA* mutations. In *BRAF-*positive patients, the density of CD4^+^ and FOXP3^+^ T cells was significantly low (p = 0.011 and p < 0.001, respectively) in the CT, whereas FOXP3^+^ T cell density was significantly high (p < 0.001) in the IM. The density of CD163^+^ macrophages in the IM was significantly high in patients with *KRAS* mutation (p = 0.038).

**Figure 5:**
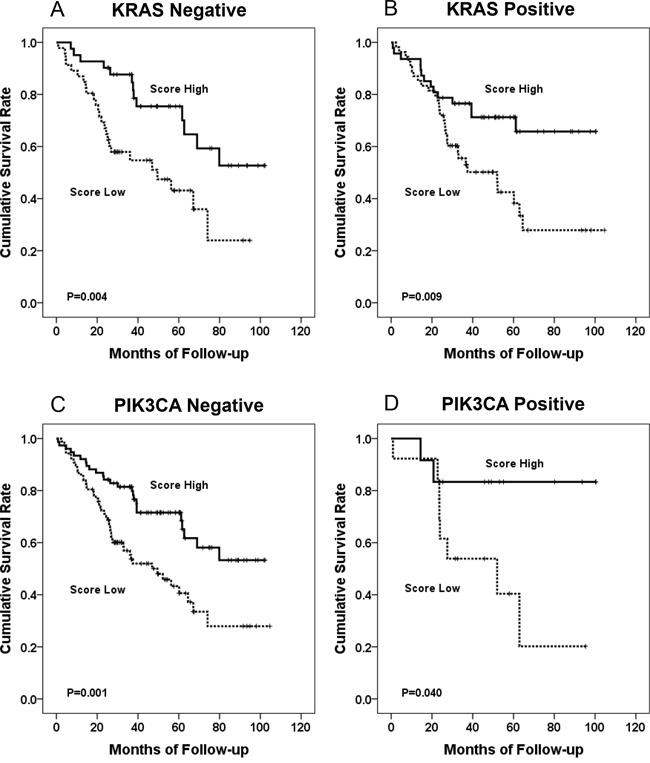
The Kaplan Meier survival curve according to IS-M model KRAS mutation-negative group **(A)**, KRAS mutation-positive group **(B)**, PIK3CA mutation-negative group **(C)**, and PIK3CA mutation-positive group **(D).**

Kaplan-Meier survival analysis revealed that *KRAS*, *PIK3CA*, and *BRAF* mutations had no significant prognostic association. In subgroup analysis, IS-M and IS-ma showed significant prognostic association regardless of the *KRAS* mutational status. The IS showed a prognostic association in *KRAS* mutation-negative group (Figure 5). IS-M and IS-ma also presented prognostic association regardless of *PIK3CA* mutational status. All three IS models showed prognostic significance in the *BRAF* mutation-negative group. None of the three IS models showed a prognostic association in the *BRAF* mutation-positive group (n = 7). Four of these 7 patients died during the follow-up period; all 4 had low IS, IS-M, and IS-ma.

### Immunoscore models assessed by using median cut-offs

We regrouped the patients into low IS and high IS groups by the median values of the continuous data of immune infiltrates. IS, IS-M, and IS-ma were recalculated by summing the scores assessed by median cut-offs. Of 196 patients, 129 (65.8%) had low IS (IS0: 14.8%, IS1: 20.9, and IS2: 30.1) and 67 (34.2%) had high IS (IS3: 20.9% and IS4: 13.3%). One hundred and twenty-five (64.7%) patients had low IS-ma (IS-ma0: 3.1%, IS-ma1: 5.7%, IS-ma2: 29.0%, and IS-ma3: 26.9%), and 68 (35.3%) patients presented with high IS-ma (IS-ma4: 23.3%, IS-ma5: 10.4%, and IS-ma6: 1.6%). Of 188 patients, 117 (62.2%) had low IS-M (IS-M0: 4.8%, IS-M1: 14.9%, IS-M2: 23.4%, and IS-M3: 19.1%), and 71 (37.8%) had high IS-M (IS-M4: 18.1%, IS-M5: 12.8%, and IS-M6: 6.9%).

When the IS was compared to the patient's clinicopathologic features, lower IS was associated with synchronous metastasis (p = 0.035). Lower IS-M was also correlated with aggressive clinicopathological features, including higher pT stage (p = 0.009), pN stage (p = 0.012), synchronous metastasis (p = 0.010), and perineural invasion (p = 0.016). There was no significant association between IS-ma and clinicopathologic features. The Kaplan–Meier survival analysis revealed that a high score of IS-M was significantly associated with good prognosis (p = 0.008), although IS and IS-ma models had no significant association (p = 0.113 and p = 0.328, IS and IS-ma respectively).

## DISCUSSION

The present study demonstrated that TILs and TAMs show significant heterogeneity in their tumor infiltration site. The density of CD3^+^ and CD8^+^ lymphocytes was higher in the IM than in the CT. The infiltration of CD3^+^, CD8^+^, and CD163^+^ immune cells was significantly different between the CT and the IM as well as between the CT and DM. Although several studies have reported the density of tumor-infiltrating immune cells of CRC patients according to the tumor sites, these reports included only two or three subsets of tumor-infiltrating immune cells or did not consider the density of tumor-infiltrating immune cells in the DM [31–33]. This study compared the density of 4 T cell subsets and 2 macrophage subsets in different sites of tumors. We also compared the density of tumor-infiltrating immune cells in primary and metastatic tumors. To our knowledge, this is the first comprehensive report of the heterogeneity of tumor-infiltrating immune cells in CRCs

In several previous studies, the protective role of T cell subsets on tumor progression has been consistently reported [34–36]. Most of studies have demonstrated that dense infiltration of CD3^+^, CD8^+^ or CD45RO^+^ lymphocytes are associated with less aggressive clinicopathological features and a better prognosis [35, 36]. In this study, our cohort was composed of patients with metastatic disease and we demonstrated the prognostic value of the IS method. Hence, TIL of tumor microenvironmental factors and the IS system could be a robust prognostic factor that is assessable for advanced CRC patients with distant metastasis.

Our results demonstrated that the IS-M model, which includes the score of two lymphocytic markers in the DM, is superior to IS or IS-ma. The conventional model designed by Galon et al. covers lymphocytic infiltrates in only the primary tumor. However, in the present study, we confirmed that tumor-infiltrating immune cells have not only heterogeneity of quantity but also distinct clinical significance in relation to the tumor location. Therefore, the immune infiltrates in metastatic lesions as well as in the primary tumor should be assessed to validate the patient's systemic immune reaction on whole tumors. This is supported our by results showing that the IS-M model was the only independent prognostic marker among the IS models in multivariate analysis.

In a recent report, Lea et al. described the limitations of the current TNM staging system in predicting the outcome of patients with CRC [20]. They suggested that the immune cell density in the stromal environment could be a better prognostic marker. This suggestion was also confirmed by Mlecnik et al [37]. Furthermore, the multivariate survival analysis conducted by Anitei et al. confirmed that the IS system has stronger prognostic value than the TNM staging system [38]. The present study demonstrated that the IS-M has a significant association with prognosis regardless of *KRAS* or *PIK3CA* mutational status. Hence, immune contexture, including immune cell density in primary and metastatic tumors, could be a reliable prognostic marker in CRC, regardless of patients' mutational status.

However, it seems that there are some challenges in applying the IS system as a prognostication factor. First, the determination of an optimal cut-off value is difficult. Galon et al. illustrated that a predetermined cut-off value should be used to score high versus low values for each marker in each location. The previous study by Galon et al., as well our study, used a maximal-chi square method to set optimal cut-off values. However, other values such as the 25^th^ percentile, median, and 75^th^ percentile could be candidates for alternative cut-off values. We calculated three IS systems using the median cut-off, and the results of survival analysis showed a similar tendency; the patients with a higher IS-M score had significantly better outcome. However, both the cut-off values calculated by the maximal-chi square method and the median values would differ according to several factors, including cohort characteristics, quality of the sample, selected area of examination, antibodies to be used, and cell counting algorithms. Thus, to set a reliable cut-off value of the density of immune infiltrates, a multicenter prospective study for the standardization of the detailed methodology is needed.

Another challenge is the selection of optimal area for density analysis. Since the density of immune infiltrates is highly heterogeneous, selection of the analyzed area could affect the results. According to Galon et al., who first suggested the IS system, the combined analysis of immune infiltrates in the CT and IM could improve the prediction of patient survival [35]. After the initial study, several studies have evaluated the IS system [37–39]. The IS system is organized and is based on the enumeration of two lymphocyte populations in the CT and IM. We also examined the immune infiltrates in the CT and IM using TMA method, similar to the previous studies. However, it is predictable that the results could be affected by the selection of the analyzed area owing to the heterogeneity of immune infiltrates. To assess this potential limitation, we investigated the density of CD3+ T cells in 4 different portions (2 CT and 2 IM areas) of 57 cases using additional TMA blocks. The median value of CD3+ T cell density was 281.72 (IQR, 160.69 – 488.91) in CT1 and 205.5174 (IQR, 109.87 – 485.96) in CT2. In IM, the median value of IM1 and IM2 were 353.67 (IQR, 208.74 – 692.79) and 331.60 (IQR, 226.95 – 455.22), respectively. We evaluated the consistency of T cell infiltrates by calculating the Pearson's correlation coefficient. The Pearson's R of T cell infiltrates in between 2 areas of CT (CT1 vs. CT2) was 0.668 (p < 0.001). Between IM1 and IM2, the Pearson's R value was 0.498 (p < 0.001). However, the statistically significant correlation of T cell densities between in CT and IM was not observed (p > 0.05). These results suggest that selection of CT and IM areas are necessary and suitable for the evaluation of immune infiltrates using TMA method. However, further studies with persuasive validation of the heterogeneity of immune infiltrates are required.

In summary, we demonstrated the regional heterogeneity of tumor-infiltrating immune cells according to the tumor location in our large cohort of advanced CRC patients with synchronous and metachronous distant metastasis. Also, the amount of immune infiltrates was also heterogeneous in relation to the metastatic organ examined. Higher infiltrates of TIL and lower infiltrates of TAM correlated with longer survival. The three IS models, IS, IS-ma, and IS-M also had prognostic significance in univariate analysis. Among the three IS methods, the IS-M model that includes TILs in the DM was an independent prognostic marker. Our results suggest that immune infiltration in the DM should be evaluated to assess the IS system for advanced CRC patients with distant metastases.

## MATERIALS AND METHODS

### Patient selection and tissue microarray construction

A total of 196 advanced CRC patients who presented with synchronous or metachronous metastases were enrolled in this study. They underwent surgical treatment for primary and metastatic disease at Seoul National University Bundang Hospital (Seongnam-si, South Korea) between 2003 and 2009. Of the 196 patients, none had received preoperative systemic therapy or radiation treatment. The patient's clinical and pathological data were obtained through medical charts and pathology reports. The patient outcomes and their survival times were collected. The patients lost to follow-up or dead from causes other than CRC were assumed as censored. The follow-up period ranged from 0.8 to 104.6 months (median, 37.3 months).

All patients with synchronous metastasis underwent adjuvant chemotherapy after the surgical resection of primary and metastatic tumors. Of the 62 patients with metachronous metastasis, 56 underwent adjuvant chemotherapy, and presented with metastatic lesions during their follow-up period. Six patients with metachronous metastasis treated with curative resection of primary cancer received no adjuvant chemotherapy after the surgical resection. Since the metastatic lesion was observed during their follow-up period, they were treated with metastasectomy and chemotherapy.

Formalin-fixed paraffin-embedded tissues from the CRCs were collected. The representative core tissues (2 mm in diameter) were used. The obtained tumor tissue included the area of CT and IM of the primary tumor as well as its related DM. Each core tissue was rearranged into tissue array blocks using a trephine apparatus (Superbiochips Laboratories, Seoul, South Korea) [40].

### Immunohistochemistry and image analysis of tumor-infiltrating immune cell

The presence of tumor-infiltrating immune cells was confirmed by immunohistochemistry using antibodies for CD3 (1:100, DAKO, Glostrup, Denmark), CD4 (RTU, Ventana, Tucson, AZ, USA), CD8 (1:100, Neomarkers, Fremont, CA, USA), FOXP3 (1:100, Abcam, Cambridge, UK), CD68 (1:100, DAKO), and CD163 (1:100, Novocastra, Newcastle, UK). Immunostaining for CD3, CD8, and FOXP3 was performed using a Bond polymer kit (Leica Microsystems) and Leica BOND-MAX autostainer (Leica Microsystems). CD4, CD68, and CD163 expression was detected immunohistochemically on a Ventana Bench mark XT autostainer (Ventana) with the OPTIVIEW universal DAB kit (Ventana).

All immunostained slides were scanned on an Aperio ScanScope^®^ CS instrument (Aperio Technologies, Inc., Vista, CA, USA) at 20 x magnifications. Each immunomarker-positive tumor-infiltrating immune cells quantified by computerized image analysis system, ImageScope™ (Aperio Technologies) (Figure1). CD3^+^, CD4^+^, CD8^+,^ and FOXP3^+^ lymphocytes were counted using the Nuclear v9 algorithm and CD68^+^ and CD163^+^ macrophages were counted using the Positive pixel count v9 algorithm. The density of immune infiltrates was obtained from the entire area of the tissue core.

### Determination of scoring system

The patients were divided into two groups by the density of each tumor-infiltrating immune cell according to each tumor location (high vs low). To set the best cut-off values, the maximal chi-square method was used related to the patient's overall survival [19, 38]. In addition, we analyzed the results according to median cut-offs. The detailed cut-off values of each variable are listed in Table 1 .

The IS is defined as a quantification system based on the combination of two markers (CD3 and CD8) in two regions [18, 19]. A high density of immunomarker-positive lymphocytes in each region was recorded as a score. We established two additional scoring models. One is the IS-M, which encompasses the density of CD3^+^ and CD8^+^ TILs in metastatic tumors. It is a summation of the score of CD3^+^ and CD8^+^ TILs in the CT, IM, and DM.

Another score model, IS-ma, is calculated by adding the score of the density of CD163^+^ TAMs in the primary tumor (CT and IM) to the IS. Thus, IS-ma includes the score of CD3^+^, CD8^+^, and CD163^+^ immune infiltrates in CT and IM. Our data showed that TAMs had an opposite prognostic correlation compared to that of TILs; a high density of TILs was recorded as score 1, but a high density of TAMs was recorded as score 0 to ensure the consistency of the scoring system. The schematic definitions of each of the three IS models are described in Figure 1E.

### Detection of mutations in *KRAS*, *BRAF*, and *PIK3CA* using real-time PCR

Hematoxylin-Eosin (HE)-stained slides of CRC tissues were reviewed by a pathologist (H.S.L). Tumor areas were identified and microscopically dissected to sections with an area of more than 1 × 1 cm and comprising more than 60% tumor cells. One or two 8-μm-thick formalin-fixed paraffin-embedded (FFPE) tumor tissue sections were de-paraffinized in xylene for 5 min at room temperature (RT), dehydrated in absolute alcohol for 5 min at RT, and air dried completely for 10 min. DNA was isolated using the Cobas DNA Sample Preparation Kit (Roche, Branchburg, NJ, USA) according to manufacturer's instructions, and the same preparation protocol was followed for all Cobas mutation kits used in this study. The concentration of the isolated DNA was measured using a NanoDrop UV spectrophotometer (Thermo Fisher Scientific, Wilmington, DE, USA), and the DNA was diluted with DNA Specimen Diluent from the Cobas 4800 Mutation Test kit (Roche) to the optimal concentration for each gene (*KRAS*, 4 ng/μL; *BRAF*, 5 ng/μL; and *PIK3CA*, 2 ng/μL). Amplification and detection were performed using an Automated Cobas X480 analyzer. The real-time PCR assay was performed to detect the mutation in codons 12, 13, and 61 of *KRAS*; the V600E *BRAF* mutation; and the mutation in exons 1, 4, 7, 9, and 20 of *PIK3CA*.

### Statistical analysis

To compare each non-continuous variable, a Wilcoxon/Mann-Whitney test or Kruskal-Wallis analysis was used. To establish the optimal cut-offs of continuous variables, the maximal chi-squared method was performed using the R program (http://cran.r-project.org/). The Kaplan-Meier method was used to examine survival outcomes and the significance of the differences between groups was compared using the log-rank test. A univariate and multivariate regression analysis was performed using Cox proportional hazards models to determine hazard ratios (HRs). P values of less than 0.05 were considered statistically significant. All statistical analysis, except for the maximal chi square test, was performed using IBM SPSS statistics 20 (Armonk, NY, USA).

## SUPPLEMENTARY MATERIALS TABLE


