# Supplementary material for: Immunoscore encompassing CD3+ and CD8+ T cell densities in distant metastasis is a robust prognostic marker for advanced colorectal cancer
Source: Oncotarget. 2016 Nov 8;7(49):81778–90. doi: 10.18632/oncotarget.13207 (PMC5348429; doi:10.18632/oncotarget.13207)
Supplement: Supplementary file 1 [file oncotarget-07-81778-s001.pdf]

Immunoscore encompassing CD3+ and CD8+ T cell densities in distant metastasis is a robust prognostic marker for advanced colorectal cancer

SUPPLEMENTARY TABLE

Supplementary Table S1: The median survival time and its comparison between two patient groups according to each immune cell markers of each respective tumor location

	Tumor location	Median survival (months)		P
		low	high	
**CD3**	Tumor center	46.93	74.1	**0.030**
	Invasive margin	60.2	-	0.059
	Distant metastasis	61.7	-	**0.013**
**CD4**	Tumor center	26.5	74.1	**0.001**
	Invasive margin	62.6	-	**0.018**
	Distant metastasis	61.7	67.1	0.723
**CD8**	Tumor center	64.5	69.0	0.900
	Invasive margin	52.2	69.0	0.144
	Distant metastasis	37.4	-	**0.002**
**FOXP3**	Tumor center	56.2	74.1	0.074
	Invasive margin	49.5	67.1	0.272
	Distant metastasis	60.2	-	**0.008**
**CD68**	Tumor center	79.8	37.9	**0.011**
	Invasive margin	67.1	62.6	0.460
	Distant metastasis	67.1	64.5	0.866
**CD163**	Tumor center	79.8	36.6	**<0.001**
	Invasive margin	64.5	-	0.981
	Distant metastasis	61.7	64.5	0.912
